# Bilateral Oval and Round Window Atresia on CT Temporal Bone: A Rare Anomaly Clinically Mimicking Otosclerosis in an Adult

**DOI:** 10.1155/2019/7457603

**Published:** 2019-12-21

**Authors:** Manzoor Ahmed, Yogesh Indrasen More, Shaik Irfan Basha

**Affiliations:** ^1^Department of Radiology, Sheikh Khalifa Medical City, Abu Dhabi, UAE; ^2^Department of ENT Surgery, Sheikh Khalifa Medical City, Abu Dhabi, UAE

## Abstract

We present a rare adult case of bilateral oval and round window atresia. Clinical and audiologic findings were suggestive of otosclerosis. High resolution CT Temporal bones showed unequivocal findings of bilateral oval and round window atresia. Atresia of these windows is a rare temporal bone anomaly. Presentation as an adult can confound the clinicians and warranting a closer look on the CT for atretic windows and subtle signs of otosclerosis in patients with conductive hearing loss.

## 1. Introduction

Isolated oval window atresia (OWA) is a rare middle ear temporal bone congenital anomaly. Multiple earlier reports have been surgical [1–3]. OWA can be confirmed on high resolution (HR) CT temporal bone (CT T-Bone). Usually there are associated middle ear findings like inferio-medial course of the facial nerve (covering the site of oval window) and malformed or displaced incus and stapes [1, 4, 5]. About 40% of the cases are bilateral [6].

**Figure 1:**
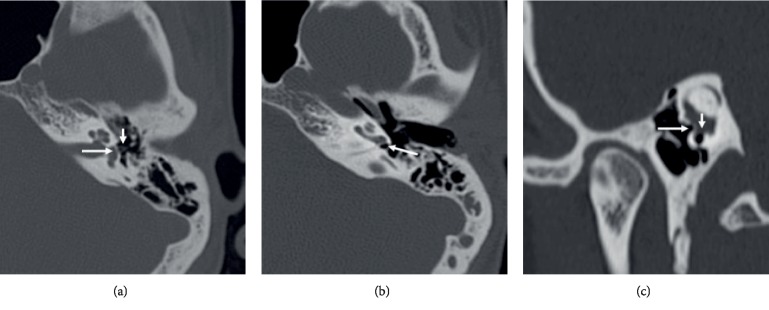
High resolution CT Temporal bone axial ((a) and (b)) and coronal oblique (Pöschl's view) imaging showing faintly visualized normal oval window defect (long arrow, (a)) with horse-shoe like stapedial crura visible in the vicinity (short arrow, (a)). Normal round window niche is marked by a small pocket of air (arrow, (b)). Both oval (long arrow, (c)) and round windows (short arrow, (c)) are well demonstrated in the same image on Pöschl's view (c).

The oval window is a small oval-shaped normal bony defect or window designed for the stapes foot-plate at the medial wall of the middle ear opening into the vestibule (Figure 1). The lateral adjacent important and reference structures include crura of the stapes and tympanic segment of the facial nerve. Ironically, presence and identification of this normal oval defect can be more subtle than vice versa. Atresia of the oval window can still be an easily over-looked finding on the CT T-Bone in a patient with conductive hearing loss. The expected finding is a band of bone instead of a window. On the same lines, there are also few other subtle and significant not infrequently overlooked findings on HR CT T-Bone e.g. Congenital or acquired bony dehiscence, Ossicular focal erosions like focal lenticular process uncal erosion, ante-fenestral otosclerosis, anomalous facial nerve course, etc. All these so called subtle findings warrant closer look and quality HR CT T-Bone study with standard reconstructions.

Round window atresia (RWA) is even more rare than OWA with few case reports [7–10]. The finding can be even overlooked on surgery. Conductive hearing loss due to RWA is theoretically related to no pressure release mechanism for inner ear fluid displaced by the stapes footplate and even total conductive hearing loss can be expected [10]. Isolated and nonsyndromic RWA is extremely rare with hearing tests mimicking otosclerosis [9]. Unlike the oval window, normal round window can be easily identified on routine angular reconstructed images of the HR CT T-Bone with a pocket or cave of air in the middle ear as a landmark for identification on axial images (Figure 1).

We report here a rare case of an adult, clinically presenting as otosclerosis until HR CT T-Bone was performed showing bilateral both oval and round window atresia. This is an extremely rare case report [11] with unequivocal manifestation of the bilateral OWA and RWA on HR CT T-Bone imaging.

## 2. Case Report

A 30-year-old female was referred for hearing loss. She presented with long-standing bilateral hearing loss. On further enquiry, the patient mentioned of having hearing loss since childhood and has been regularly using hearing-aids. She did not have any significant history of ear infections or ear trauma. She denied any significant past medical, surgical or family history. Clinical assessment shows bilateral normal ear canals, both sides tympanic membranes were within normal limits.

**Figure 2:**
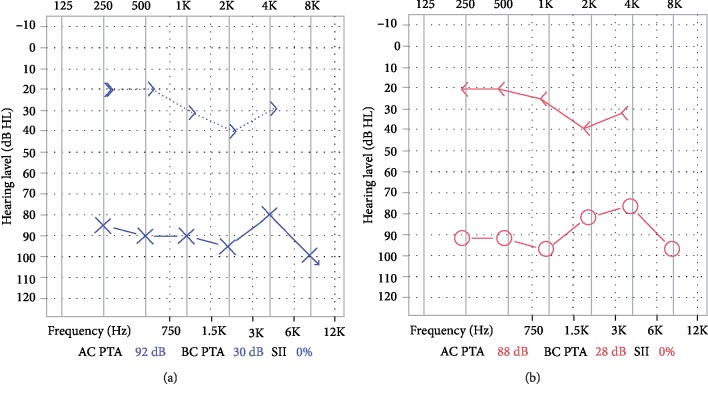
Pure tone audiometry (PTA) of left ear (a) and right ear (b) showing bilateral severe conductive hearing loss. Note bilateral intact unmasked bone conduction (BC) (marked by arrowheads) and profound flat air conduction (AC) loss (marked by X for left and circle for right ear) across all frequencies resulting in significant air-bone conduction (ABC) gap.

Hearing assessment performed in our department showed a conductive hearing loss in both ears. Bone conduction (BC) in both ears almost normal with a dip at 2 KHz frequency. Air conduction (AC) and air-bone gap was abnormal. There was a large air-bone gap of 60 dB (AC 88 dB and BC 28 dB) in the right ear while 62 dB (AC 92 dB and BC 30 dB) in the left ear (Figure 2). Speech reception was 90 dB in right and 85 dB in left. Tympanometry and stapedial reflex was within normal limits for both ears.

**Figure 3:**
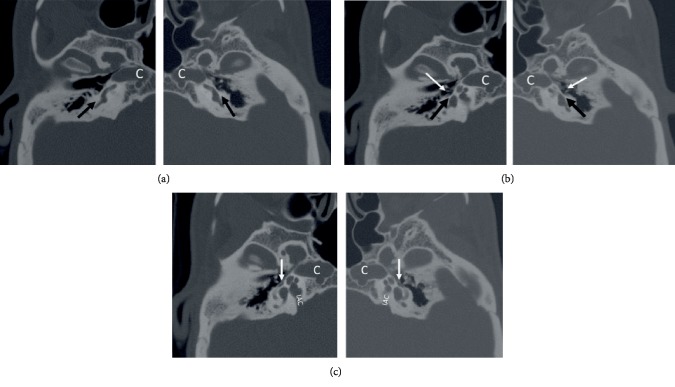
(a) High resolution axial CT Temporal bone images at the level of round window (a), stapes and oval window (b), and tympanic facial nerve (c). Absence of bilateral round ((a), dark arrows) and oval windows ((b), dark arrows) with tympanic facial nerve covering the site of oval window (C, arrows). Stapedial anomalies present with aplastic crura on the right and nearly intact left stapes except hypoplastic anterior crus (white arrows, (b)). Note skull base dysmorphism with abnormal orientation of internal auditory canal (IAC) and kissing carotid canals (marked as C in Figures 3(a)–3(c)).

**Figure 4:**
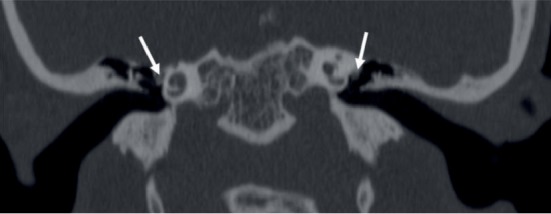
Coronal high resolution CT Temporal bone image showing bilateral dehiscent tympanic segments of facial nerve (arrows), abutting the lateral wall of the bony labyrinth due to its inferio-medial anomalous course.

Based on clinical and audiology assessment, we suspected middle ear-ossicular chain pathology, most probably otosclerosis given the chronologic age of the patient. Patient underwent High Resolution CT Temporal Bone (HR CT T-Bone) imaging mainly to confirm otosclerosis. CT showed the following findings on both the right and left temporal bones:

- Absence of round windows (Figure 3(a), arrows) with sclerotic bone and adjacent posterior mesotympanic dysmorphism with atretic sinus tympani.
- Absence of oval window (Figure 3(b), dark arrows) with retained thick bony plate without a defect into the vestibule. Note aplastic crura of the stapes on the right and relatively well developed stapes on the left side except the hypoplastic anterior crus (Figure 3(b), white arrows).
- Tympanic facial nerve anterio-medial positioning covering the site of the oval window (Figure 3, arrows) as well as showing dehiscence (Figure 4, arrows)
- Skull base dysmorphism with near-sagittal orientation of the internal auditory canal (IAC) and kissing carotid canals (annotated with C in Figures 3(a)–3(c)).
- No HR-CT evidence of even subtle changes of the ante-fenestral and or the peri-cochlear otosclerosis.

The patient has normal speech development while currently her speech reception thresholds (SRT) are about 90 dB which is classified as profound hearing loss, this fact explains the progressive nature of this condition. Patient opted for hearing aids. Currently the patient is rehabilitated with high power hearing aids and coping well due to well preserved cochlea.

## 3. Discussion

We presented a rare adult case of bilateral oval and round window atresia presenting clinically as a case suggestive of otosclerosis. The case underscores the utility of HR CT T-Bone as a crucial preoperative aid in identification of atretic oval window as well as the rarer coexisting finding of round window atresia. This case will fall under Class 4 congenital middle ear anomalies.

A classification system was developed by Teunissen and Cremers [12] to analyze the findings. Class 1 comprises ears with congenital isolated stapes ankylosis. Class 2 comprises ears with congenital stapes ankylosis in combination with a congenital anomaly of the ossicular chain. Class 3 comprises ears with congenital anomalies of the ossicular chain and at least a mobile stapes footplate. Class 4 comprises ears with aplasia or severe dysplasia of the oval window or round window. This warrants us to understand the embryologic basis of oval and round window atresia.

The embryologic development of the oval window is closely related to the development of the second branchial arch structures in about 5^th^–7^th^-week of development. The most important structure in this relationship is the facial nerve. Facial nerve along with lenticular process of the incus and stapes super-structure develop from the 2^nd^ branchial arch. However, it is the contact of stapes which will incite the development of the oval window, which is derived from the otic capsule. Two theories have been proposed to explain OWA [13, 14]: (a) Failure of fusion of stapes with the primitive vestibule resulting in nondevelopment of cleavage plane between the superiorly located lateral semicircular canal and inferior promontory canal, and so the oval window cannot form. (b) Interposition of the facial nerve between stapes blastema and prospective oval window preventing its development. Both theories indicate the consequential anterior and inferior positioning of the facial nerve basically covering the expected site of the oval window. Round window on the other hand is not covered by ossicular apparatus and appears bare on imaging. During surgery, it is partially covered by an overhanging ridge from the promontory (“subiculum promontorii”) which needs to be removed for better exposure of the round window [15]. The window is covered by a membrane which bulges in response to tap on the stapes indirectly indicating mobile stapes and corresponds to the perilymph motion of the cochlea. Originating from the otic capsule, round window can have variable developmental morphology [16] including atresia which is important for surgery as round window is the preferred access to the inner ear for implantation of the cochlear implant.

Patients with OWA typically present at a younger age (unlike our case) with moderate to severe conductive hearing loss. The differential diagnosis can be broad mainly into middle ear acquired and congenital abnormalities and possibly inner ear anomalies. As the sound wave energy moves the ossicles-stapes footplate at the oval window, the round window membrane moves in an opposite phase to the movements of the oval window dissipating the sound energy. This mechanism allows the incompressible fluid in the cochlea to move causing movement of the basilar membrane which stimulates the inner ear hair cells that forms the basis of hearing. Bone conduction hearing involves direct stimulation of the cochlea which is housed firmly in the temporal bone [17]. Isolated obliteration of the oval window leads to approximately 40 dB conductive hearing loss, however our patient had >60 dB conductive hearing loss.

Simultaneous fixation of the round and the oval window leads to cancellation of their differential movements leading to a high degree of conductive hearing loss.

The HR CT T-Bone is the modality of choice to diagnose OWA and RWA as well as an indispensable preoperative tool. CT will show absence of the oval shaped or rounded shaped niches on either axial, coronal or Pöschl view. Instead, there is obliteration of these windows by thick plates of the otic capsule.

Axial images need to be assessed at three (caudo-cranial) levels of interest: (a) Round window level (Figure 3(a)): showing lack of the round niche and adjacent air pocket or cave as well as malformed posterior-medial wall of the middle ear specifically the nondeveloped medially located sinus tympani, (b) Stapes level (Figure 3(b)): The horseshoe-shaped stapes is usually malformed including absence of one or both crura (like our case). Adjacent incus may be malformed, (c) Tympanic facial nerve level (Figure 3(c)): horizontal segment of facial nerve is mal-positioned to cover the site of oval window niche and even the malformed stapes may be attached to the facial nerve. Coronal and Pöschl (oblique coronal) views have the advantage to demonstrate both oval and round windows in the same plane and image (Figure 1).

**Figure 5:**
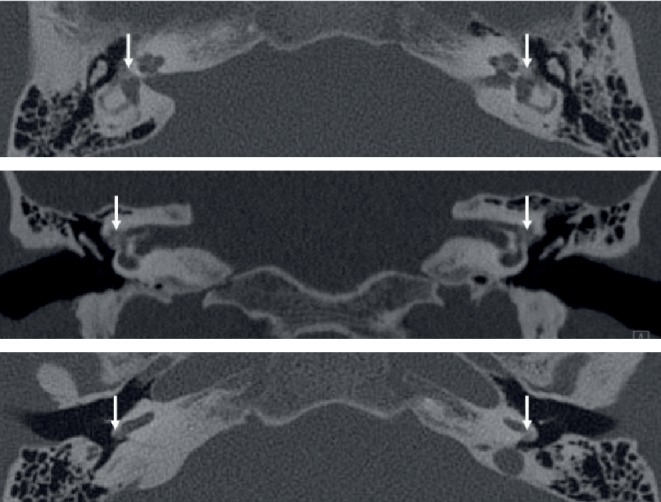
High resolution CT Temporal axial (top and bottom row) and coronal images (middle row) in another case showing ante-fenestral otosclerosis manifesting as ill-defined lucencies (arrows, top and middle row). Note even involvement of round windows (arrows, bottom row).

In cases of normal CT imaging, congenital stapes fixation (CSF) and otosclerosis need to be included in the differential diagnosis of the middle ear causes of conductive hearing loss. CSF can be confused with OWA. Patients with CSF have normal stapes on CT however, without the development of the annular ligament causing footplate ankylosis. Grade 1 (ante-fenestral) otosclerosis usually have subtle finding of the ante-fenestral lucency (Figure 5) and can mimic OWA like our adult patient as otosclerosis is a disease of middle ages and bilateral in majority of cases [18]. Otosclerosis can even cause obliteration of the oval window mimicking atresia especially in cases of heaped up osteo-spongiotic changes [18].

Management options in such rare cases are essentially limited to hearing aids for rehabilitation. Given the progressive nature of the clinical condition, our patient will need regular adjustment of the hearing aids. She is a suitable candidate for bone-conduction hearing aids which work on the principle of directly stimulating the cochlea bypassing the conductive pathway. Surgery for bone-conducting hearing aids (Bone anchored hearing aids BAHA, Bone bridge-implant) can be contemplated in such cases. Surgical correction for OWS itself can be difficult, as there are few landmarks for the vestibule, and the exposed facial nerve is at risk for injury. Sterkers et al. [19] described drilling a fenestra above the region of the oval window and then placing a piston with optimal results.

In summary, the HR CT T-Bone is an essential tool in patients with bilateral conductive hearing loss. Apparent normal scan warrants careful and critical multiplanar evaluation of the ossicular continuity and fixation, presence of the oval and round windows, otosclerotic lucencies, and associated positioning of the tympanic 7^th^ nerve. Our case is an extremely rare congenital anomaly of bilateral oval and round window atresia unmasked on the HR CT. There are few management options and usually sufficed to optimization of the hearing aids.

## References

[1] Homeer H., Kunst H., Verbist B., Cremers C. (2012). Congenital oval or round window anomaly with or without abnormal facial nerve course: surgical results for 15 ears. *Otology & Neurotology*.

[2] Vincent R., Wegner I., Derks L. S., Grolman W. (2016). Congenital oval or round window malformations in children: surgical findings and results in 17 cases. *The Laryngoscope*.

[3] Booth T. N., Vezina L. G., Karcher G., Dubovsky E. C. (2000). Imaging and clinical evaluation of isolated atresia of the oval window. *AJNR American Journal of Neuroradiology*.

[4] Zeifer B., Sabini P., Sonne J. (2000). Congenital absence of the oval window: radiologic diagnosis and associated anomalies. *AJNR American Journal of Neuroradiology*.

[5] Yang F., Liu Y., Sun J., Li J., Song R. (2016). Congenital malformation of the oval window: experience of radiologic diagnosis and surgical technique. *European Archives of Oto-Rhino-Laryngology*.

[6] Hughes A., Danehy A., Adil E. (2016). Case 226: oval window atresia. *Radiology*.

[7] Clifford A. R., Fagan P. A., Doust B. D. (1990). Isolated congenital round window absence. *The Journal of Laryngology and Otology*.

[8] Pappas D. G., Pappas D. G., Hedlin G. (1998). Round window atresia in association with congenital stapes fixation. *The Laryngoscope*.

[9] Borrmann A., Arnold W. (2007). Nonsyndromal round window atresia: an autosomal dominant genetic disorder with variable penetrance?. * European Archives of Oto-Rhino-Laryngology*.

[10] Linder T. E., Ma F., Huber A. (2003). Round window atresia and its effect on sound transmission. *Otology & Neurotology*.

[11] Vercruysse J. P., Casselman J., De Foer B., Somers T., Offeciers E. (2006). Congenital bilateral oval and round window aplasia with a hypoplastic stapes. *Otology & Neurotology*.

[12] Teunissen E. B., Cremers W. R. (1993). Classification of congenital middle ear anomalies Report on 144 ears. *Annals of Otology, Rhinology and Laryngology*.

[13] Jahrsdoerfer R. A. (1988). Embryology of the facial nerve. *The American Journal of Otology*.

[14] Gerhardt H. J., Otto H. D. (1981). The intratemporal course of the facial nerve and its influence on the development of the ossicular chain. *Acta Oto-laryngologica*.

[15] Luers J. C., Hüttenbrink K. B. (2016). Surgical anatomy and pathology of the middle ear. *Journal of Anatomy*.

[16] Tóth M., Alpár A., Patonay L., Oláh I. (2006). Development and surgical anatomy of the round window niche. *Annals of Anatomy—Anatomischer Anzeiger*.

[17] Voss S. E., Rosowski J. J., Peake W. T. (1996). Is the pressure difference between the oval and round windows the effective acoustic stimulus for the cochlea?. *The Journal of the Acoustical Society of America*.

[18] Purohit B., Hermans R., Op de Beeck K. (2014). Imaging in otosclerosis: a pictorial review. *Insights into Imaging*.

[19] Sterkers J. M., Sterkers O. (1988). Surgical management of congenital absence of the oval window with malposition of the facial nerve. *Advances in Oto-rhino-laryngology*.

